# An evaluation of a FluoroSpot assay as a diagnostic tool to determine SARS-CoV-2 specific T cell responses

**DOI:** 10.1371/journal.pone.0258041

**Published:** 2021-09-30

**Authors:** Sara M. Mangsbo, Sebastian Havervall, Ida Laurén, Robin Lindsay, August Jernbom Falk, Ulrika Marking, Martin Lord, Marcus Buggert, Pierre Dönnes, Gustaf Christoffersson, Peter Nilsson, Sophia Hober, Mia Phillipson, Jonas Klingström, Charlotte Thålin

**Affiliations:** 1 Department of Pharmaceutical Biosciences, Science for Life Laboratory, Uppsala University, Uppsala, Sweden; 2 Department of Clinical Sciences, Karolinska Institute, Danderyd Hospital, Stockholm, Sweden; 3 Department of Medical Cell Biology, Science for Life Laboratory Uppsala University, Uppsala, Sweden; 4 Department of Protein Science Division of Protein Technology, KTH Royal Institute of Technology, Stockholm, Sweden; 5 Department of Medicine, Centre for Infectious Medicine, Karolinska Institute, Stockholm, Sweden; 6 SciCross AB, Skövde, Sweden; 7 Department of Protein Science Division of Affinity Proteomics, KTH Royal Institute of Technology, Science for Life Laboratory, Stockholm, Sweden; Waseda University: Waseda Daigaku, JAPAN

## Abstract

Numerous assays evaluating serological and cellular responses have been developed to characterize immune responses against SARS-CoV-2. Serological assays are both cost- and time-effective compared to cellular assays, but cellular immune responses may provide a diagnostic value to determine previous SARS-CoV-2 infection in seronegative individuals. However, potential cross-reactive T cell responses stemming from prior encounters with human coronaviruses (HCoVs) may affect assay specificity. In this study, we evaluated the specificity and sensitivity of a SARS-CoV-2 IFN-γ Release Assay (IGRA) based on the FluoroSpot method employing commercially available SARS-CoV-2-specific peptide pools, as well as an in-house designed SARS-CoV-2 peptide pool restricted to 5 amino acid stretches or less aligning with endemic HCoVs. Blood samples were obtained from healthcare workers (HCW) 5–6 months post SARS-CoV-2 spike (S) IgG and nucleocapsid (N) IgG dual seroconversion (n = 187) and HCW who had been S IgG and N IgG dual seronegative at repeated occasions, including the current sampling time point (n = 102). In addition, samples were obtained 4 to 5 months post infection from 55 polymerase chain reaction (PCR)-confirmed COVID-19 patients. Assay specificity and sensitivity were calculated with serology as a reference standard for HCW. The in-house generated peptide pool displayed a specificity of 96.1%, while the commercially available peptide pools displayed specificities of 80.4% and 85.3%, respectively. Sensitivity was higher in a cohort of previously hospitalized COVID-19 patients (96.4% and 84.0% for the commercially available peptide pools and 92.7% for the in-house generated peptide pool) compared to the HCW cohort (92.0% and 66.8% for the commercially available peptide pools and 76.0% for the in-house generated peptide pool). Based on these findings, the individual diagnostic value of T cell immune responses against SARS-CoV-2 currently appears to be limited but remain an important research tool ahead.

## Introduction

Since the outbreak of SARS-CoV-2 in Wuhan in 2019 and the subsequent global pandemic, major efforts have been invested into large, population-based immunological surveys assessing long-term immune responses after COVID-19 contraction. The vast majority of COVID-19 cases seroconvert (91–99%) [1–4] and neutralizing IgG levels remain relatively stable for at least eight months [2, 5]. The majority of individuals infected with COVID-19 generate SARS-CoV-2-specific T cell responses [5–7]. In an eight months follow-up study, levels of SARS-CoV-2 specific CD4+ T cells remained stable over time, whereas circulating CD8+ T cells were suggested to have a shorter half-life [7]. However, the magnitude and duration of the cellular response have been difficult to clearly assess due to the presence of cross-reactive T cell responses owing to previous encounters with endemic human coronaviruses (HCoVs), thus affecting the assay’s specificity. Importantly, many peptide pools spanning the SARS-CoV-2 proteins include peptides overlapping with regions of endemic HCoVs, resulting in cross-reactive T cell responses to SARS-CoV-2 in pre-pandemic samples as shown by Mateus et al. [8] as well as Le Bert et al. [9] and our recent data [5], which provides a challenge when developing diagnostic tests based on T cell responses. Nonetheless, the large public interest for assays determining immunity against SARS-CoV-2 on an individual basis, along with more or less sensitive serological assays and limited PCR testing capacity early in the pandemic, has led to the launch of cellular immune response assays targeting the general public. Herein, we characterized the cellular response to a set of SARS-CoV-2 peptide pools in two serologically well-defined cohorts collected during the autumn of 2020. These analyses allowed us to evaluate the sensitivity and specificity of a FluoroSpot-based IGRA for cellular diagnostic purposes.

## Material and methods

### Study participants

Blood samples were obtained from the ongoing COMMUNITY (COVID-19 Biomarker and Immunity) study [5, 10–12]) which investigates long-term immunity after COVID-19 infection and vaccination. 2149 HCW and 118 COVID-19 patients were enrolled at Danderyd Hospital, Stockholm, Sweden, between April 15^th^—June 8^th^ 2020. Blood samples are obtained every four months and SARS-CoV-2 S and N-specific IgG are analyzed by multiplex antigen bead array (FlexMap3D, Luminex Corp) as previously described [10, 13]. For this sub-study, blood samples were collected in October 2020 and the data set includes 333 individuals, while the specific analysis herein include 187 HCW 5–6 months after S and N IgG dual seroconversion (5–6 months post mild to moderate COVID-19), and 102 HCW who were S and N IgG dual seronegative at all time points during the study period (i.e. at study inclusion in April-June 2020, at the four-month follow-up in August-September 2020 and at the sampling time point in October 2020). Individuals that seroconverted later in the study are not part of the final analysis. Blood samples were also obtained from 55 convalescent and previously hospitalized COVID-19 patients at the four-month follow-up (4–5 months post severe infection). A sufficient number of cells were obtained to run all peptide pool stimulations on 187 seropositive HCW and 102 seronegative HCW. For the patients, the S1 spanning pool (peptide pool 166, consisting of 166 peptides in total) was used in 50 of the 55 individuals due to limited cell numbers. The study complied with the declaration of Helsinki, and informed consent was obtained by all participants. The study protocol was approved by the Stockholm Ethical Review Board (Dnr 2020–01653).

### FluoroSpot analysis

Peripheral blood mononuclear cell (PBMC) isolation was performed using a Ficoll density gradient together with SepMate™ tubes, according to the manufacturer’s instructions (StemCell, Canada) and cells were cryopreserved in FBS (Gibco) with 10% DMSO (Tocris, England). SARS-CoV-2-specific T cell reactivity was evaluated by the dual IFN-γ and IL-2 (data from the IL-2 readout are not evaluated herein) FluoroSpot (Mabtech) using a commercial S1 peptide pool, referred to as P166, spanning the S1 domain of the S protein, a commercial peptide mixed pool, referred to as P47, with peptides derived from the S protein, N protein, membrane protein and the open reading frame (ORF)-3a and ORF-7a proteins (both from Mabtech) and an in-house designed SARS-CoV-2-specific pool, referred to as P16, with no more than 5 amino acid stretches aligning with endemic HCoVs previously used in a whole-blood assay assessing SARS-CoV-2 specific T cell responses [5]. The peptide sequences for the in-house pool as well as the overlapping sequencing with endemic HCoVs, SARS and MERS for all peptide pools used are presented in the S1 and S2 Tables. Anti-CD3 (CD3-2, Mabtech) was used as a positive control and DMSO only (matching the volume of the added peptide/DMSO) as a negative control. The cryopreserved PBMCs were thawed and rested overnight in complete RPMI 1640 with GlutaMax™ medium (Gibco), supplemented with 10% FBS (Gibco) and 100 Units of penicillin-streptomycin (Gibco). FluoroSpot plates pre-coated with anti-IFN-γ (1-D1K, Mabtech) and anti-IL-2 (MT2A91/2C95, Mabtech) were washed three times with PBS and blocked with complete medium overnight at 4°C. Cells were harvested and plated in duplicates with 2.5x10^5^ cells/well for peptide stimulations, and 0.5x10^5^ cells/well for anti-CD3 stimulation. SARS-CoV-2 specific peptide pools were added at a concentration of 2 μg/ml for each individual peptide. Cells were stimulated for 24h at 37°C with 5% CO_2_. The plates were washed five times with PBS before incubation with diluted (1:200) anti-IFN-γ (7-B6-1-BAM) and 1:500 anti-IL-2 (MT8G10-biotinylated) antibodies (all from Mabtech) for 2h at RT, followed by 1h incubation with secondary fluorophore-conjugated antibodies, anti-BAM 490 (1:200), and streptavidin-550 (1:200). Lastly, the fluorophore enhancer was added for 10 min. Between each step, the plates were washed five times with PBS, except after the addition of the fluorophore enhancer. The plates were read using a Mabtech IRIS and spots were analyzed using Mabtech Apex software 1.1. The threshold was set for a binary T cell response criterion based on a 2-fold or greater increase in the spot-forming units (SFU) above the individual persons own control sample (blood incubated with vehicle buffer only). Individuals with a negative control SFU value below 10 were only scored positive if they had a peptide-induced memory T cell response (SFU) that was > the [negative control +10]. The individual scoring method was used to align with a test setup allowing for individual T cell response scoring for public use.

### Serology analyses

The serological analysis was performed using a multiplex antigen bead array in a high throughput 384 well plate format as previously described [10, 13]. IgG reactivity was measured towards spike trimers comprising the perfusion-stabilized, S-glycoprotein ectodomain and the C-terminal domain of the nucleocapsid-protein. The viral proteins were linked to the surface of color-coded magnetic microbeads (Luminex Corp). IgG reactivity was detected using a phycoerythrine-conjugated, goat anti-human IgG (H10104, Invitrogen) and measured as mean fluorescence intensity (MFI) per bead ID in a FlexMap3D system (Luminex Corp). The antigen-specific threshold for seropositivity was defined as the mean MFI plus 6 SD of 12 negative controls included for each assay run. An individual was scored as seropositive if both antigens passed this threshold. Utilizing this definition and 2090 negative controls sampled before 2020 along with 331 positive controls sampled at least 17 days after positive PCR or symptom onset led to a calculated assay sensitivity of 99.7% and a specificity of 100% [10, 13].

### Statistical analysis

Data analysis was performed in R (4.0.3) using the epiR (2.0.33) and caret (6.0.86) statistical packages.

## Results

### IFN-γ responses in seronegative health care workers

**Fig 1:**
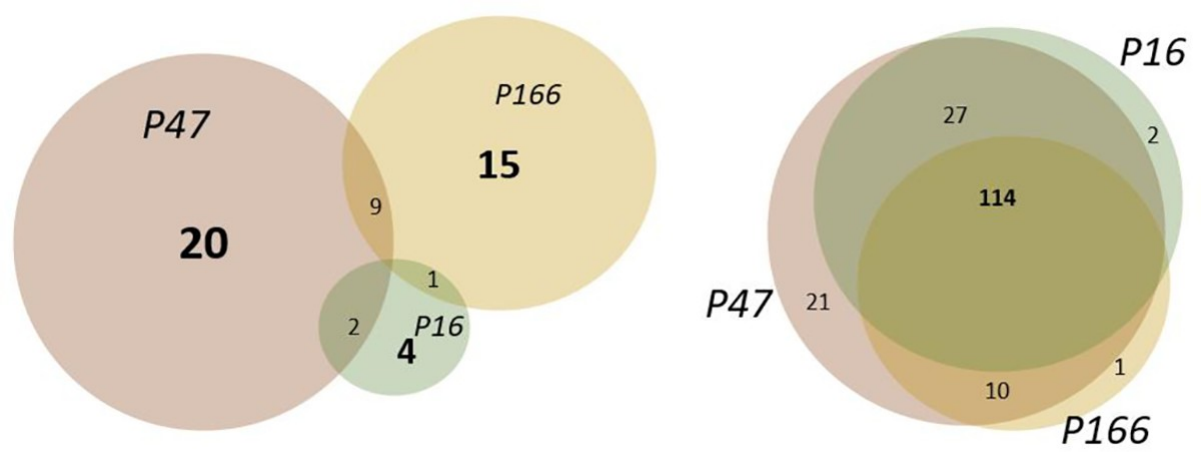
Venn diagram displaying (left) the seronegative individuals (total n = 102 tested) that scored T cell positive (numbers in bold for each peptide pool) on the T cell test. The individuals displaying dual positive scoring are noted in each overlapping circle. In the right Venn diagram, the number of individuals with a positive T cell test in the seropositive group (total n = 187 tested) is presented. The bold number 114 represents the number of individuals with a positive scoring for all peptide pools. Dual and single positive test results are also noted in the right Venn diagram.

We first investigated the SARS-CoV-2-specificity of the T-cell responses elicited towards each SARS-CoV-2 peptide pool by determining the responses generated in samples obtained from the HCW who remained seronegative at all time points during the study period and where IFN-γ responses to all peptide pools were available (n = 102, Table 1). For P47, 20/102 individuals displayed a positive scoring resulting in a specificity of 80.4%. For P166, 15/102 individuals displayed a positive scoring resulting in a specificity of 85.3%. For the in-house generated P16 pool, 4/102 individuals tested positive, resulting in a specificity of 96.1%. The two commercially available SARS-CoV-2 peptide pools (P47 and P166) include peptides with potential overlap with endemic HCoVs, which may affect SARS-CoV-2 specificity. Thus, a double positive criterion was also assessed, where an individual was required to test positive for both of these peptide pools. Using this criterion, 9/102 HCW scored positive, increasing specificity to 91.3%. Table 3 presents an overview of the specificity for each pool including the 95% confidence intervals and Fig 1 represents an overview of the positive scoring and the overlap of peptide pools within the seronegative HCWs (left figure).

**Table 1:** HCW.

P47	T cell positive	T cell negative	Total
IgG positive	172	15	187
IgG negative	20	82	102
Total	192	97	289
P166	T cell positive	T cell negative	Total
IgG positive	125	62	187
IgG negative	15	87	102
Total	140	149	289
P16	T cell positive	T cell negative	Total
IgG positive	143	44	187
IgG negative	4	98	102
Total	147	142	289
P47+P166 combined analysis	T cell positive	T cell negative	Total
IgG positive	124	63	187
IgG negative	9	93	102
Total	133	156	289

### IFN-γ responses in seropositive health care workers and PCR-confirmed previously hospitalized COVID-19 convalescents

Disease severity has been suggested to correlate with the magnitude of the cellular immune memory response and may therefore also affect sensitivity when immune responses are analyzed after recovery ([6] and our unpublished data). We, therefore, used samples obtained from both seropositive HCW 5–6 months post mild to moderate disease (n = 187) and COVID-19 patients 4–5 months post severe disease (n = 55 (n = 50 for the P166 peptide pool)), Tables 1 and 2. Using the P47 SARS-CoV-2 peptide pool, 172/187 seropositive HCW and 53/55 COVID-19 patients scored positive on the T cell assay, with a resulting sensitivity of 92.0% and 96.4% respectively. Using the P166 SARS-CoV-2 peptide pool, 125/187 seropositive HCW and 42/50 COVID-19 patients scored positive on the T cell assay, corresponding to a sensitivity of 66.8% and 84.0% respectively. Using the in-house generated SARS-CoV-2 P16 peptide pool, 143/187 seropositive HCW and 51/55 COVID-19 patients scored positive on the T cell assay, resulting in a sensitivity of 76.5% and 92.7% respectively. Requiring positive scoring on both commercial pools decreased sensitivity, resulting in 124/187 seropositive HCW and a sensitivity of 66.3% for HCWs and 84.5% for patients. Table 3 presents an overview of the sensitivities for each pool including the 95% confidence intervals See also Fig 1 for an overview of the positive scoring and the overlap between peptide pools within the seropositive HCWs.

**Table 2:** Patients.

Patient data, PCR and IgG positive	T cell positive	T cell negative	Total
P47	53	2	55
P166	42	8	50
P16	51	4	55
P47+P166	42	8	50

**Table 3:** Point estimates and 95% confidence intervals for respective peptide pool.

%	P47	P166	P16	P47+P166
Sensitivity	92 (87, 95)	67 (60, 74)	76 (70, 82)	66 (59, 73)
Specificity	80 (71, 88)	85 (77, 92)	96 (90, 99)	91 (84, 96)
Positive predictive value	90 (84, 94)	89 (83, 94)	97 (93, 99)	93 (88, 97)
Negative predictive value	85 (76, 91)	58 (50, 66)	69 (61, 76)	60 (51, 67)
Accuracy	88 (84, 91)	73 (68, 78)	84 (79, 87)	75 (70, 80)

### Assay accuracy

Predictive values were calculated on the HCW cohort solely to reflect the robustness of the test if to be used as a diagnostic test for the general population. The accuracy of the test was 87.9% using the P47 peptide pool, 73.4% using the P166 peptide pool and 83.4% using the P16 peptide pool. For the combined analysis utilizing the responses to both commercial pools, the accuracy was 75.1%. The positive predictive values were 89.6% when using the P47 peptide pool, 89.3% when using the P166 peptide pool and 97.3% when using the P16 peptide pool respectively. For the combined peptide pool scoring using the responses to both the P47 and P166 peptide pools, the positive predictive value (PPV) was 93.2%. The negative predictive value (NPV) was 84.5% when using the P47 peptide pool, 58.4% when using the P166 peptide pool and 69.0% when using the P16 peptide pool. When the combined positive response criteria for both commercially available pools were used, the negative predictive value was 59.6%. See Table 3 for a summary of the PPV, NPV and accuracy values including the 95% confidence intervals.

## Discussion

We assessed the sensitivity and specificity of a FluoroSpot SARS-CoV-2 T-cell assay using three different SARS-CoV-2 peptide pools. Our results indicate that the S1 spanning peptide pool (P166) leads to a high degree of false-positive responses, despite a high degree of uniqueness in amino acid sequences differentiating the domain from endemic HCoVs, and does not provide an ideal sensitivity in individuals post mild COVID-19 disease. The broader SARS-CoV-2 peptide pool (P47) provides high sensitivity, but at the cost of low specificity, likely related to an immunodominant peptide region present in the majority of HCoVs (own unpublished data and [9]). We also show that it is possible to provide a higher specificity, albeit to a cost of a lower sensitivity in individuals with mild to moderate COVID-19, by selecting peptides without epitopes overlapping with endemic HCoVs. Taken together, our data suggest that it is possible to, by careful peptide selection, generate a peptide pool resulting in a high specificity (>95%) for COVID-19 diagnostic purposes. However, this specificity is lower than what is aimed at in serological assays that often achieve a specificity greater than 99.5%. The relatively low sensitivity of the FluoroSpot-based IGRA assay furthermore limits clinical implementation with the aim to identify COVID-19 convalescents when serology and PCR have not been performed within a timeframe that allows for a positive test result. As next-generation sequencing and artificial intelligence are now entering the arena of diagnostic tools, we may in the future see advances when it comes to T cell receptor sequencing to predict previous infections. A step towards such a scenario was recently taken with the emergency use authorization by the U.S. Food and Drug Administration of the T-Detect COVID assay from Adaptive Biotechnologies [14].

Limitations to our study include the possibility that a portion of HCW may have had a mild COVID-19 disease without seroconverting, resulting in an underestimation of specificity in our analyses. However, considering the now well-established high rate of seroconversion and longevity of circulating S IgG following also mild COVID-19 [1–5, 10], our frequent blood samplings initiated early in the pandemic, along with the high sensitivity of the serological assay used in this study, we consider this risk to be low. The sensitivity is highly dependent on disease severity but may also be HLA-dependent. As such, the number of peptides and epitopes included in peptide pools influences sensitivity. However, our findings show a sensitivity of 93% using the in-house generated SARS-CoV-2 P16 peptide pool, the peptide pool with the fewest peptides, in the cohort of PCR-confirmed previously hospitalized COVID-19 convalescents, arguing against a limitation in HLA coverage. The reduced sensitivity of the assays found in the HCW cohort thus likely reflects disease severity and time from exposure.

In summary, our findings do not support the use of a diagnostic FluoroSpot IGRA-based T cell test to diagnose prior COVID-19 disease.

## Supporting information

S1 Table The following peptides are included in the in-house generated SARS-CoV-2 specific peptide pool. (DOCX)

S2 Table Overview of the length and specific amino acids of the peptide sequences from the peptide pools that overlap with endemic coronaviruses as well as SARS and MERS. (DOCX)

S1 File (XLSX)
