# Supplementary material for: An evaluation of a FluoroSpot assay as a diagnostic tool to determine SARS-CoV-2 specific T cell responses
Source: PLoS One. 2021 Sep 30;16(9):e0258041. doi: 10.1371/journal.pone.0258041 (PMC8483319; doi:10.1371/journal.pone.0258041)
Supplement: S1 Table — (DOCX) [file pone.0258041.s001.docx]

**Supplementary table I**

The following peptides are included in the in-house generated SARS-CoV-2 specific peptide pool

| Protein | Position | Sequence |
| --- | --- | --- |
| Spike | 166-180 | CTFEYVSQPFLMDLE |
|  | 261-285 | GAAAYYVGYLQPRTFLLKYNENGTI |
|  | 365-394 | YSVLYNSASFSTFKCYGVSPTKLNDLCFTN |
|  | 450-469 | NYLYRLFRKSNLKPFERDIS |
|  | 495-514 | YGFQPTNGVGYQPYRVVVLS |
|  | 562-586 | FQQFGRDIADTTDAVRDPQTLEILD |
|  | 606-622 | NQVAVLYQ**G**VNCTEVPV |
|  | 606-622 | NQVAVLYQ**D**VNCTEVPV |
| NP | 44-64 | GLPNNTASWFTALTQHGKEDL |
|  | 130-140 | IIWVATEGALN |
|  | 5-21 | GPQNQRNA**P**RITFGGPS |
|  | 5-21 | GPQNQRNA**L**RITFGGPS |
| M | 141-158 | GAVILRGHLRIAGHHLGR |
|  | 172-188 | TSRTLSYYKLGASQRVA |
| NSP3 | 1569-1584 | FASFYYVWKSYVHVVD |
| NSP7 | 36-50 | HNDILLAKDTTEAFE |
