# Supplementary material for: An evaluation of a FluoroSpot assay as a diagnostic tool to determine SARS-CoV-2 specific T cell responses
Source: PLoS One. 2021 Sep 30;16(9):e0258041. doi: 10.1371/journal.pone.0258041 (PMC8483319; doi:10.1371/journal.pone.0258041)
Supplement: S2 Table — (DOCX) [file pone.0258041.s002.docx]

**Supplementary table II**

Overview of the length and specific amino acids of the peptide sequences from the peptide pools that overlap with endemic coronaviruses as well as SARS and MERS are listed below:
